# Construction and validation of a nomogram for predicting survival in elderly patients with cardiac surgery

**DOI:** 10.3389/fpubh.2022.972797

**Published:** 2022-10-19

**Authors:** Tonghui Xie, Qi Xin, Xing Zhang, Yingmu Tong, Hong Ren, Chang Liu, Jingyao Zhang

**Affiliations:** ^1^Department of Hepatobiliary Surgery, The First Affiliated Hospital of Xi'an Jiaotong University, Xi'an, China; ^2^Department of Thoracic Surgery, The First Affiliated Hospital of Xi'an Jiaotong University, Xi'an, China; ^3^Department of Surgical ICU (SICU), The First Affiliated Hospital of Xi'an Jiaotong University, Xi'an, China

**Keywords:** cardiac surgery, elderly patients, risk factor, 1-year all-cause mortality, nomogram

## Abstract

**Background:**

In recent years, the number of elderly patients undergoing cardiac surgery has rapidly increased and is associated with poor outcomes. However, there is still a lack of adequate models for predicting the risk of death after cardiac surgery in elderly patients. This study sought to identify independent risk factors for 1-year all-cause mortality in elderly patients after cardiac surgery and to develop a predictive model.

**Methods:**

A total of 3,752 elderly patients with cardiac surgery were enrolled from the Medical Information Mart for Intensive Care III (MIMIC-III) dataset and randomly divided into training and validation sets. The primary outcome was the all-cause mortality at 1 year. The Least absolute shrinkage and selection operator (LASSO) regression was used to decrease data dimensionality and select features. Multivariate logistic regression was used to establish the prediction model. The concordance index (C-index), receiver operating characteristic curve (ROC), and decision curve analysis (DCA) were used to measure the predictive performance of the nomogram.

**Results:**

Our results demonstrated that age, sex, Sequential Organ Failure Assessment (SOFA), respiratory rate (RR), creatinine, glucose, and RBC transfusion (red blood cell) were independent factors for elderly patient mortality after cardiac surgery. The C-index of the training and validation sets was 0.744 (95%CI: 0.707–0.781) and 0.751 (95%CI: 0.709–0.794), respectively. The area under the curve (AUC) and decision curve analysis (DCA) results substantiated that the nomogram yielded an excellent performance predicting the 1-year all-cause mortality after cardiac surgery.

**Conclusions:**

We developed a novel nomogram model for predicting the 1-year all-cause mortality for elderly patients after cardiac surgery, which could be an effective and useful clinical tool for clinicians for tailored therapy and prognosis prediction.

## Introduction

Population aging has become a major conundrum over the last few decades. Previous research indicates that the global population over 60 has increased from 10% in 2000 to 21.8% in 2050 (1). Furthermore, cardiovascular disease affects more than 70% of people over 60 (2), accounting for the increase in elderly patients undergoing cardiac surgery. At present, cardiac surgery is one of the most common surgical operations. The death rate continues to be high as a result of the lengthy operation, severe surgical trauma, and frequent postoperative complications. It is worth noting that due to the gradual degeneration of organ functions and more organic comorbidities, the risk of mortality is significantly higher in elderly patients with cardiac surgery (3, 4).

A number of previous scoring systems, including the European System for Cardiac Operative Risk Evaluation (EuroSCORE II) (5) and the North American Society of Thoracic Surgeons (STS) (6), have offered crucial clinical reference values in predicting postoperative mortality related to cardiac surgery. These scoring systems, however, include a large number of variables, are relatively difficult to use, and primarily focus on preoperative indicators, limiting their clinical application. Moreover, several studies have revealed bias in the above scoring systems' ability to predict a patient's risk of death following cardiac surgery (7, 8). Recently, some single factors, such as the level of postoperative serum creatinine (9), mean body temperature within 24 h after cardiac surgery (10), the preoperative lymphocyte-to-monocyte ratio (LMR) (11), and RBC transfusion (12), have been associated with adverse outcomes following cardiac surgery. Nevertheless, given the specificity of the older cardiac patient population, there is a lack of systematic models for elderly patients with cardiac surgery to predict the occurrence of 1-year all-cause mortality.

Nomograms are widely used to predict clinical adverse events, given that they enable visualization of statistical prediction models to a single numerical estimate of the probability of an event, such as death, based on the patient's conditions (13). Therefore, the contribution of this article includes two aspects. (1) Identify risk factors that can predict 1-year all-cause mortality in elderly patients with cardiac surgery; (2) To develop a feasible, effective nomogram model to evaluate the incidence of 1-year all-cause mortality in elderly patients with cardiac surgery, which will provide clinicians with effective individualized intervention information ahead of time.

## Materials and methods

### Data source

The study was a retrospective cohort study, and all data were obtained from Medical Information Mart for Intensive Care III (MIMIC-III) database (14), an open-access public database containing information on over 40,000 critically ill patients treated at Beth Israel Deaconess Medical Center in Boston, Massachusetts, between 2001 and 2012. The database's application was approved by the Beth Israel Deaconess Medical Center and Beth Israel Deacons Medical Center's institutional review committees (Approval Code: 28572693). Individual informed consent was not required since patient-related information in the database was anonymous.

### Patient selection

The following patients were selected from the MIMIC-III database: (1) Patients who underwent cardiac surgery, including CABG and Valvular surgery; (2) The majority of developed nations worldwide recognized the chronologic age of 65 as a definition of “elderly,” according to the World Health Organization's definition of older person. Therefore, we included the post-cardiac surgery population aged ≥65 years; (3) Patients without a past medical history of malignant tumor; (4) The full records of serum markers within 24 h of ICU admission; (5) Complete clinical data available.

### Data extraction

The Structured Query Language (SQL) was applied to query and acquire all clinical data, and pgAdmin4 served as the PostgreSQL administrative platform. The extracted data mainly included: (1) Demographic characteristics: age, sex. (2) vital signs: heart rate (HR), respiratory rate (RR), and mean arterial pressure (MAP). (3) laboratory results within 24 h after cardiac surgery: creatinine, glucose, hematocrit, hemoglobin, platelet, potassium, activated partial thromboplastin time (APTT), international normalized ratio (INR), prothrombin time (PT), sodium, blood urea nitrogen (BUN) and white blood cell (WBC). (4) Score system: Sequential Organ Failure Assessment (SOFA). (5) RBC transfusion. The variables included were the mean values within 24 h of ICU admission. The main outcome of this study was the 1-year all-cause mortality.

### Statistical analysis

The entire dataset was divided into two groups: the training set (60%, *n* = 2,252) and the validation set (40%, *n* = 1,500). The training set was used to construct the nomogram prediction model, while the validation set was used to verify the accuracy of the nomogram. The chi-square test was used to compare clinical data characteristics between the training and validation sets. In the training set, variable distributions were assessed using Shapiro-Wilk tests. Continuous variables with normally distributed distributions were expressed as means and standard deviation (SD), while non-normally distributed variables were reported as the median and interquartile range (IQR). For normally distributed variables, the student's *t*-test was used to compare continuous variables, and the Mann–Whitney U-test was used for non-parametric data. The chi-square, or Fisher's exact test, was used to compare categorical variables. The Least Absolute Shrinkage and Selection Operator (LASSO) regression analysis is applied to a selected training set's most potent predictive characteristic. According to LASSO regression analysis results, the following variables were selected for multivariate logistic regression analysis: age, sex, SOFA, HR, RR, creatinine, glucose, platelet, potassium, and RBC transfusion. Finally, a nomogram model for 1-year all-cause mortality in elderly patients who have undergone cardiac surgery is constructed using multivariate logistic analysis based on training sets. The nomogram's differentiation ability was assessed using the C-index and an area under the receiver operating characteristic curve (AUC-ROC) analysis. A relatively corrected C-index was obtained *via* bootstrapping validation (1,000 Bootstrap Resamples). In addition, the clinical net benefit of the prediction model was assessed using decision curve analysis (DCA).

Statistical analysis was performed using R version 4.1.3 software equipped with the “glmnet,” “rms,” and “rmda” packages and SPSS version 26.0. A *P*-value < 0.05 was statistically significant.

## Results

### Baseline characteristics

**Figure 1:**
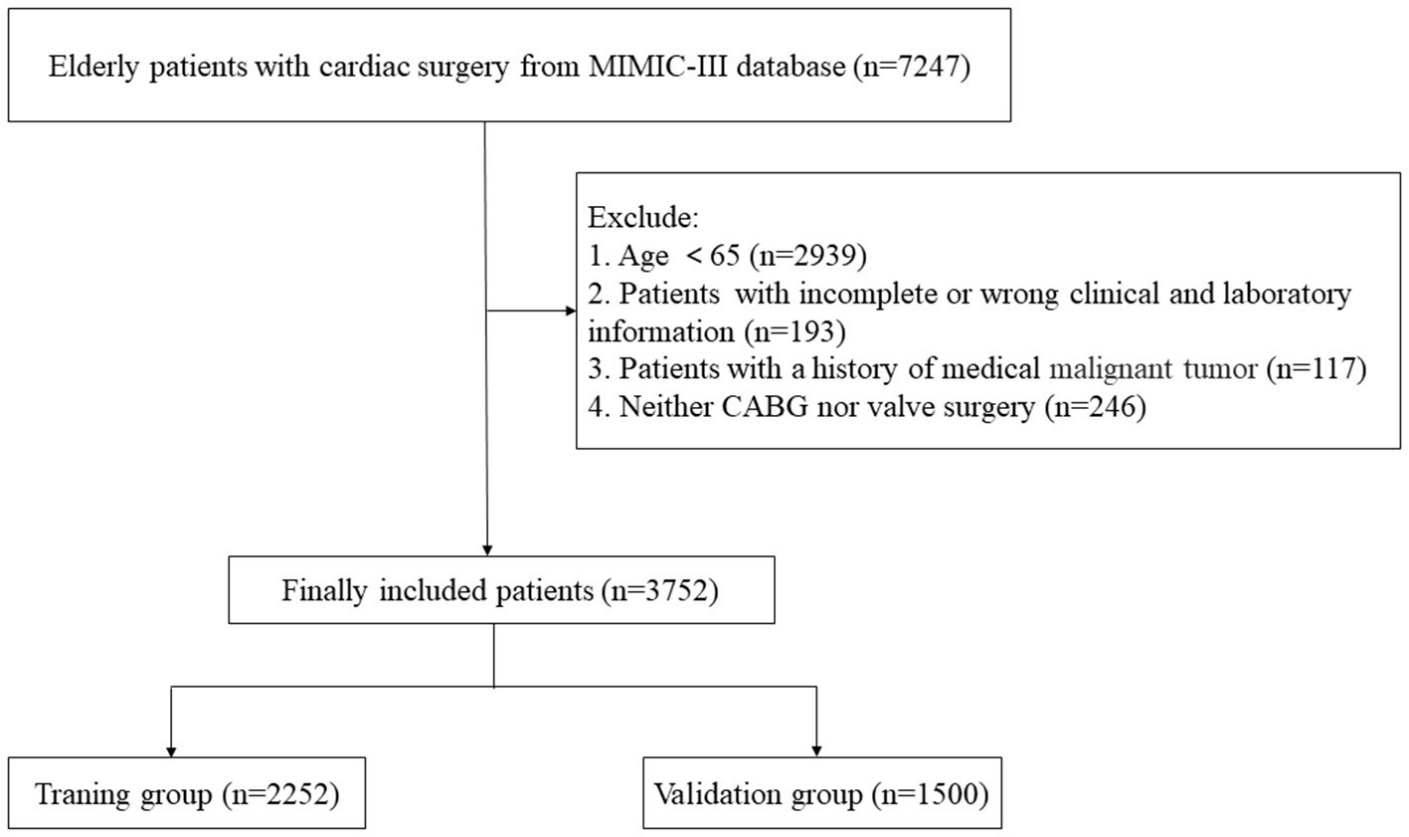
The flowchart of patient selection.

A total of 7,247 patients that underwent cardiac surgery between 2001 and 2012 were initially enrolled from the MIMIC-III dataset. 3,495 patients that met the exclusion criteria were excluded from the study, leaving 3,752 patients to be analyzed (Figure 1). Of these, 349 (9.3%) patients died within 1 year of cardiac surgery. All patients were randomly assigned to the training set (*n* = 2,252) or the validation set (*n* = 1,500), with a theoretical ratio of 6:4. Table 1 shows the demographic and clinical features of patients that underwent cardiac surgery in the training and validation sets. The patients in the training set had a median age of 75 years (interquartile range, 70 to 80), and 35.8% (n = 806) were female. The validation set included 557 females (37.1%) with a median age of 74 (interquartile range 69 to 80 years). In the training and validation sets, 1-year all-cause mortality was observed in 8.7% and 10.1% of elderly patients that underwent cardiac surgery. There was no significant difference between the training and validation sets, indicating that their baseline was comparable (*p* > 0.05).

**Table 1:** Demographic characteristics of the elderly patients with cardiac surgery.

**Variables**	**Total**	**Training**	**Validation**	***P-*value**
	***n* = 3752**	***n* = 2252**	***n* = 1500**	
**Death**, ***n*** **(%)**				0.169
No	3,403 (90.7)	2,055 (91.3)	1,348 (89.9)	
Yes	349 (9.3)	197 (8.7)	152 (10.1)	
**Age**	75 (70,80)	75 (70,80)	74 (69,80)	0.355
**Sex, n (%)**				0.422
Female	1,363 (36.3)	806 (35.8)	557 (37.1)	
Male	2,389 (63.7)	1,446 (64.2)	943 (62.9)	
**Vital signs**				
HR, times/min	83.0 (78.0,89.0)	83.0 (78.0,89.0)	83.0 (77.0,89.0)	0.109
RR, times/min	17.0 (15.0,19.0)	17.0 (15.0,19.0)	17.0 (15.0,19.0)	0.566
MAP, mmHg	74.0 (70.0,78.0)	74.0 (70.0,78.0)	74.0 (70.0,78.0)	0.256
**Laboratory test**				
Creatinine, mg/dL	0.9 (0.7,1.1)	0.9 (0.7,1.1)	0.9 (0.7,1.1)	0.929
Glucose, mg/dL	132.7 (121.6,147.2)	132.6 (121.7,146.7)	133.1 (121.3,148.3)	0.641
Hematocrit, %	29.3 (27.2,31.9)	29.3 (27.2,31.8)	29.2 (27.3,32.0)	0.803
Hemoglobin, g/dL	9.9 (9.1,10.8)	9.9 (9.1,10.8)	9.9 (9.1,10.9)	0.852
Platelet, 10^9^/L	159.0 (129.0,197.0)	157.0 (127.6,196.0)	160.5 (130.7,198.5)	0.074
Potassium, mmol/L	4.4 (4.2,4.6)	4.4 (4.1,4.6)	4.4 (4.2,4.6)	0.772
APTT, s	37.0 (31.9,45.2)	37.0 (31.8,44.9)	36.9 (31.9,45.7)	0.663
INR	1.4 (1.3,1.5)	1.4 (1.3,1.5)	1.4 (1.3,1.5)	0.220
PT, s	14.9 (14.1,15.8)	14.8 (14.1,15.8)	14.9 (14.1,15.8)	0.297
Sodium, mmol/L	137.5 (136.0,139.0)	137.5 (136.0,139.0)	137.5 (136.0,139.0)	0.830
BUN, mg/dL	17.0 (13.3,22.0)	17.0 (13.0,22.0)	17.0 (13.5,22.0)	0.502
WBC, 10^9^/L	11.8 (9.4,14.7)	11.9 (9.5,14.7)	11.8 (9.4,14.8)	0.773
**Score system**				
SOFA	5.0 (3.0,7.0)	5.0 (3.0,7.0)	5.0 (3.0,7.0)	0.762
**RBC transfusion**, ***n*** **(%)**				0.796
No	1,700 (45.3)	1,016 (45.1)	684 (45.6)	
Yes	2,052 (54.7)	1,236 (54.9)	816 (54.4)	
**Surgery type**, ***n*** **(%)**				
CABG	2,355 (62.8)	1,402 (62.3)	953 (63.5)	0.448
Valvular surgery	1,397 (37.2)	850 (37.7)	547 (36.5)	

HR, heart rate; RR, respiratory rate; MAP, mean arterial pressure; APTT, activated partial thromboplastin time; INR, international normalized ratio; PT, prothrombin time; BUN, blood urea nitrogen; WBC, white blood cell; SOFA, sequential organ failure assessment; RBC transfusion, red blood cell transfusion; CABG, coronary artery bypass grafting.

### LASSO regression analysis

**Figure 2:**
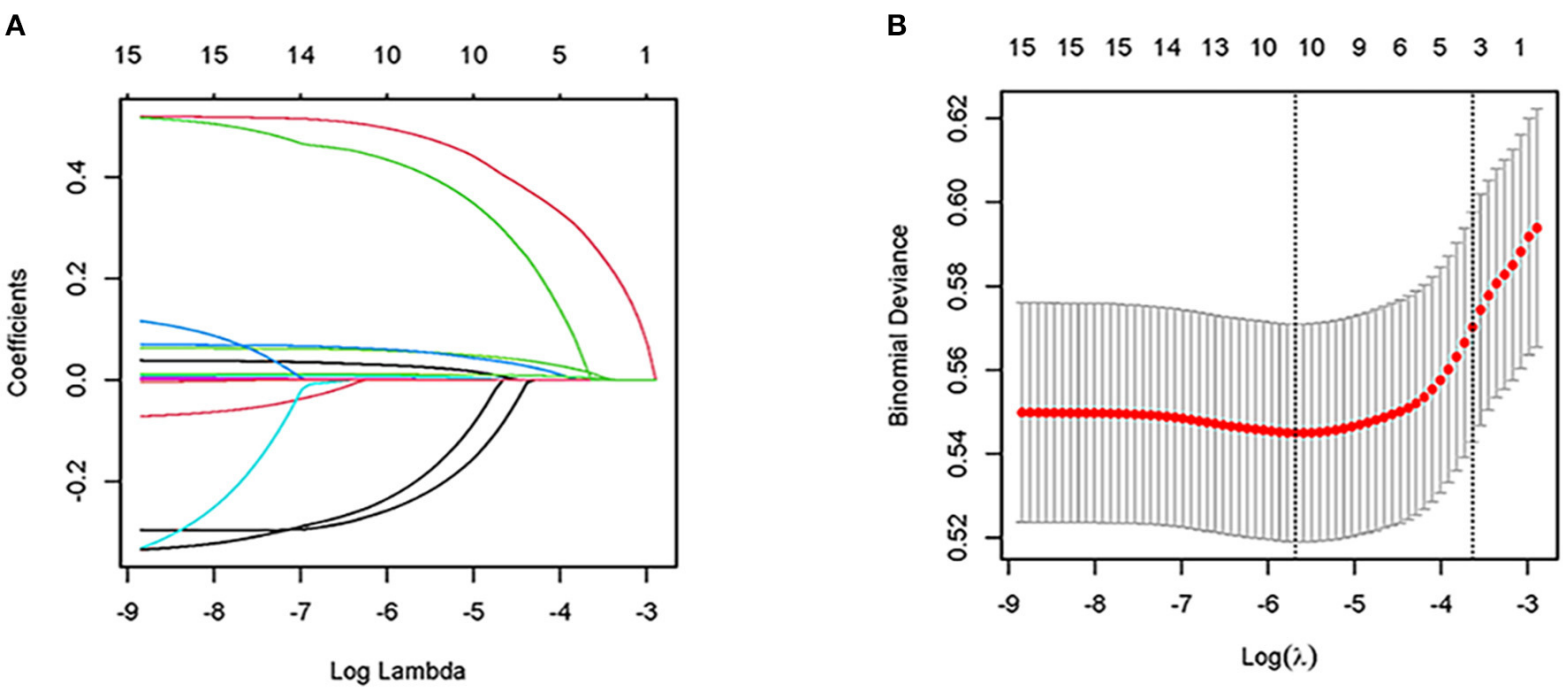
Clinical feature selection by LASSO. **(A)** Plot of LASSO coefficient profiles of the 20 features. The log (lambda) sequence was plotted against a coefficient profile plot. There were 10 features with non-zero coefficients generated by the ideal lambda (λ = 0.003403811); **(B)** 10-fold cross-validation for LASSO model parameter adjustment. The binomial deviation curve was displayed with log (lambda). The minimum criteria and its one standard error were used to construct dotted vertical lines at the optimal values (the 1-SE criteria).

A total of 20 related variables (age, sex, HR, RR, MAP, creatinine, glucose, hematocrit, hemoglobin, platelet, potassium, APTT, INR, PT, sodium, BUN, WBC, SOFA and RBC transfusion) in our study were originally input into the LASSO regression method by 10-fold cross validation to determine the prognostic factors of 1-year all-cause mortality in elderly patients who have undergone cardiac surgery. Finally, we chose ten variables with the best lambda (λ = 0.003403811): age, sex, SOFA, HR, RR, creatinine, glucose, platelet, potassium and RBC transfusion (Figure 2).

### Multivariate logistic regression analysis of independent factors for 1-year all-cause mortality

To identify independent factors predicting 1-year all-cause mortality in elderly patients who have undergone cardiac surgery, multivariate logistic regression analysis was used to screen the 10 LASSO-selected predictors. Then, the following factors were associated with a significantly elevated risk of 1-year all-cause mortality: age (OR = 1.063, 95% CI = 1.039–1.088, *p* < 0.001), SOFA (OR = 1.067, 95% CI = 1.008–1.130, *p* = 0.026), sex (OR = 0.709, 95% CI = 0.516–0.973, *p* = 0.033), RR (OR = 1.053, 95% CI = 1.000–1.108, *p* = 0.048), creatinine (OR = 1.649, 95% CI = 1.409–1.930, *p* < 0.001), glucose (OR = 1.013, 95% CI = 1.008–1.017, *p* < 0.001), and RBC transfusion (OR = 1.661, 95% CI = 1.173–2.353, *p* = 0.004) (Table 2).

**Table 2:** Multivariate logistic regression analyses of independent predictors for 1-year all-cause death in the elderly patients with cardiac surgery.

**Variables**	**OR**	**95%CI**	***P–*value**
Age	1.063	1.039–1.088	< 0.001
SOFA	1.067	1.008–1.130	0.026
RR, times/min	1.053	1.000–1.108	0.048
Creatinine, mg/dL	1.649	1.409–1.930	<0.001
Glucose, mg/dL	1.013	1.008–1.017	<0.001
Male	0.709	0.516–0.973	0.033
RBC transfusion	1.661	1.173–2.353	0.004

SOFA, sequential organ failure assessment; RR, respiratory rate; RBC transfusion, red blood cell transfusion.

### The development of nomogram model in the training set

**Figure 3:**
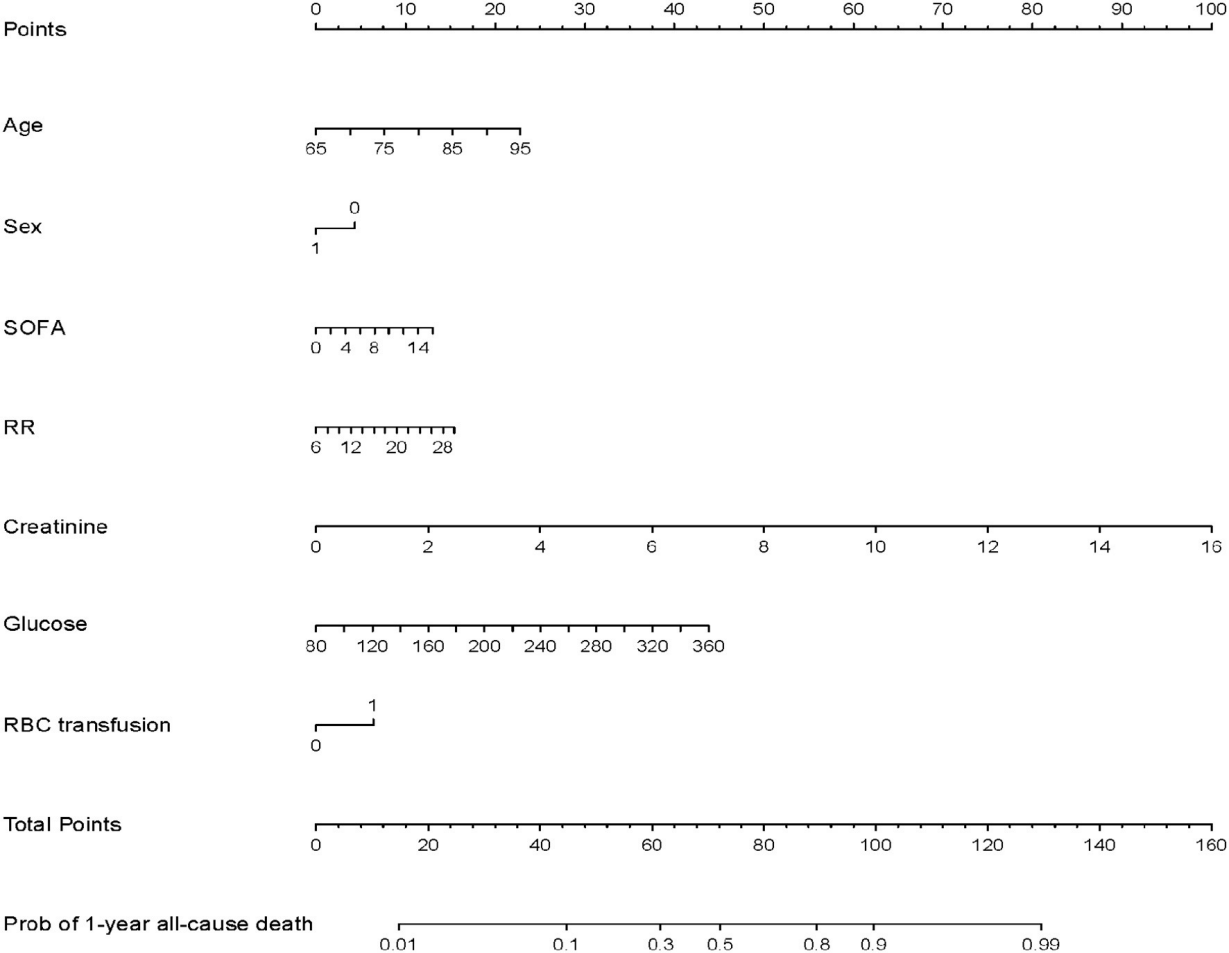
Nomogram model for predicting 1-year all-cause mortality in elderly patients with cardiac surgery.

The seven variables selected for multivariate logistic regression analysis (age, SOFA, sex, RR, creatinine, glucose, surgery type and RBC transfusion) were used to construct a nomogram for predicting the risk of 1-year all-cause mortality in elderly patients with cardiac surgery. As shown in Figure 3, the nomogram showed that creatinine had the greatest impact on patient prognosis, followed by glucose and age. The probability of 1-year all-cause mortality can be estimated by calculating the total number of points from the vertical line of the variable to the point axis.

### Validation of the nomogram model

**Figure 4:**
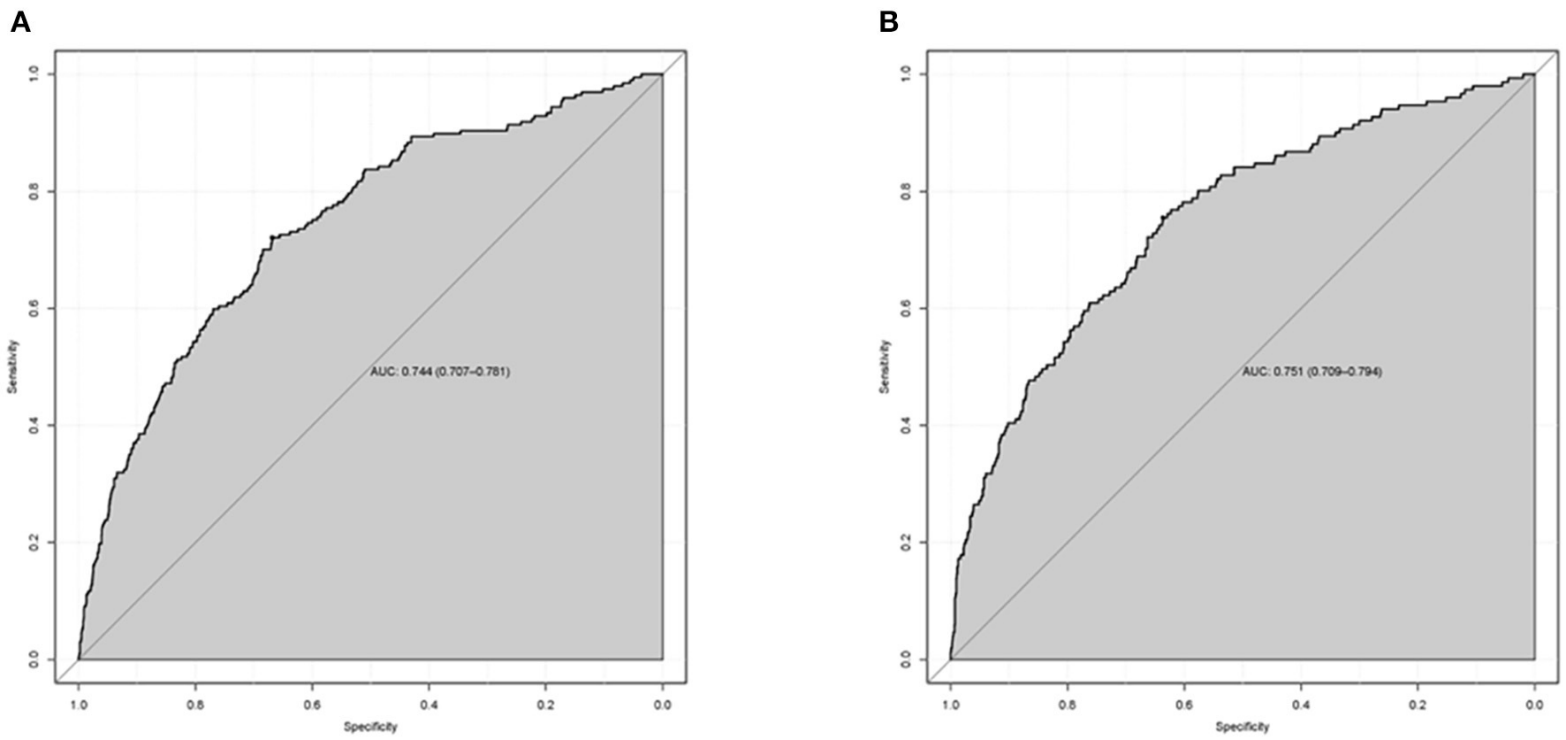
The area under the receiver operating characteristic (ROC) curves (AUCs) of the nomogram for predicting 1-year all-cause mortality in training set **(A)** and validation set **(B)**. The ROC curve was drawn according to the predictive value of the nomogram and the 1-year all-cause mortality in elderly patients after cardiac surgery.

**Figure 5:**
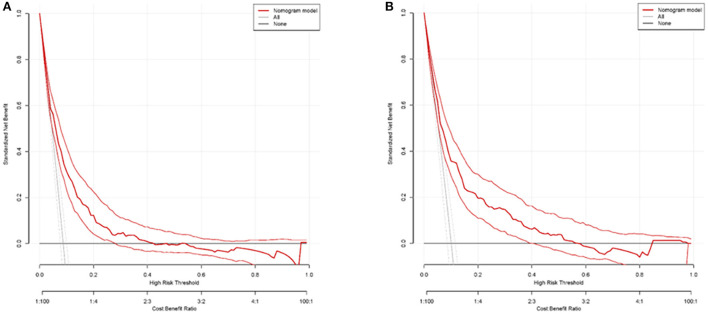
Decision curve analysis (DCA) of the nomogram model for predicting 1-year all-cause mortality in training set **(A)** and validation set **(B)**. The abscissa shows the threshold probability of 1-year all-cause mortality prediction, and the ordinate represents the net benefits of benefits and hazards. The black parallel horizontal line above the abscissa indicates that none of the patients died, and the net benefit is 0. The gray line indicates that the 1-year all-cause mortality occurred in all patients.

**Figure 6:**
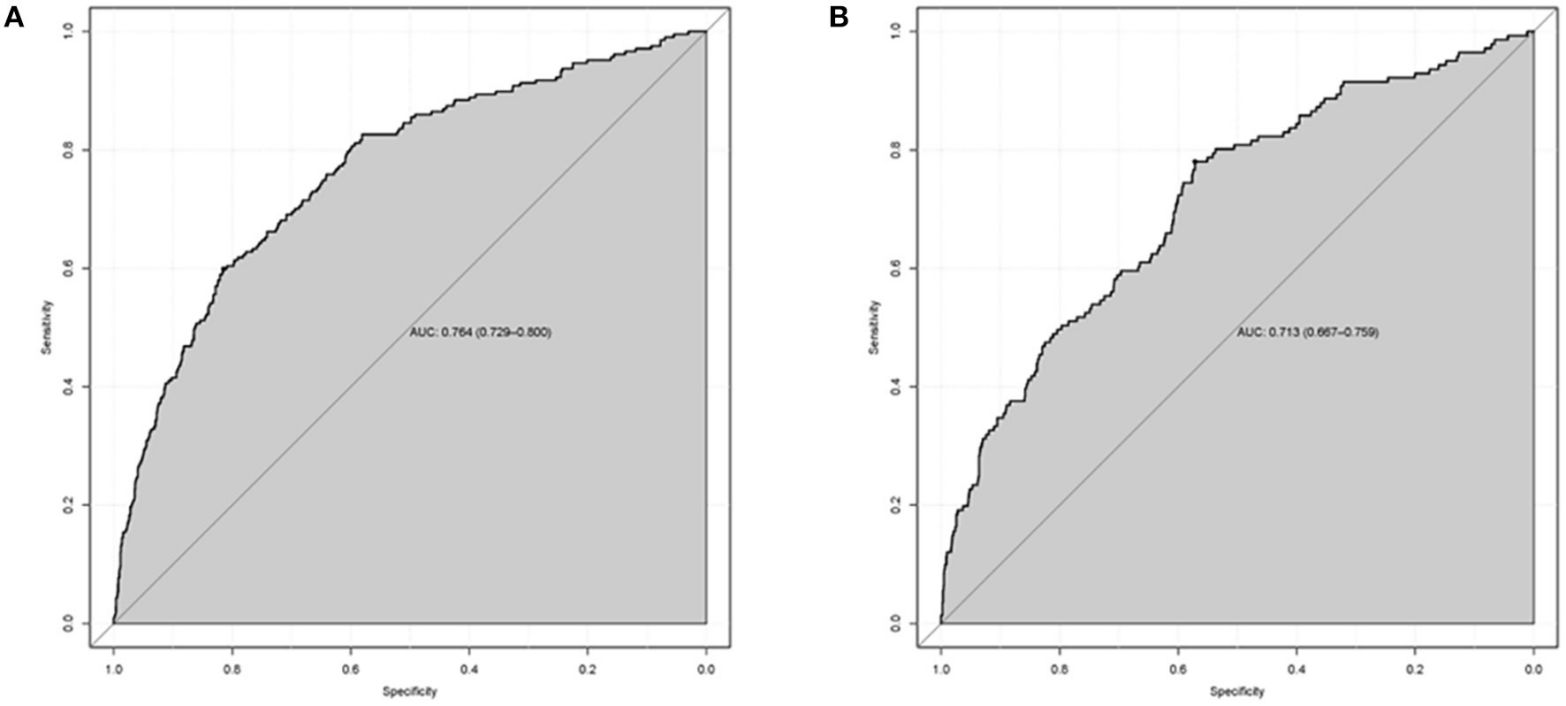
The area under the receiver operating characteristic (ROC) curves (AUCs) of the nomogram for predicting 1-year all-cause mortality in CABG group **(A)** and Valvular surgery group **(B)**.

The C-index for the training and validation sets was 0.744 (95%CI: 0.707–0.781) and 0.751 (95%CI: 0.709–0.794), respectively, consistent with the ROC curve analysis results (Figure 4). These findings indicate that the nomogram model has good predictive performance for the 1-year all-cause mortality. DCA revealed that the nomogram had a superior overall net benefit within the wide and practical ranges of threshold probabilities, as shown in Figure 5, indicating a high potential clinical utility. Moreover, to further validate the model's power for prediction, we also performed subgroup analysis in accordance with various types of surgery. The results show that the C-index and AUC of the CABG groups were 0.765 (95%CI: 0.730–0.800) and 0.764 (95%CI: 0.729–0.800), respectively. The C-index and AUC of the Valvular surgery groups were 0.714 (95%CI: 0.668–0.760) and 0.713 (95%CI: 0.667–0.759), respectively (Figure 6).

## Discussion

Population aging has been accompanied by the rapid growth of elderly populations and a series of health-related problems, becoming a major concern. Age is well-established to be associated with adverse outcomes in postoperative cardiac patients, particularly in the elderly (3, 4, 15). In elderly patients with insufficient organ reserve capacity, the body is in a chronic inflammatory state, which is the cause of a high incidence of perioperative adverse events. In addition, compared to younger people, older people have a higher percentage of comorbidities and frequently have serious cardiac disease. Therefore, the risk of cardiac surgery in older patients is significantly higher than that in younger patients. This study concentrated on the potential risk factors for 1-year all-cause mortality in elderly patients after cardiac surgery in order to early identify critically ill individuals and provide timely, individualized treatment. Our research showed that the 1-year all-cause mortality of patients with cardiac surgery was 9.3%, consistent with the literature (16). Moreover, our research found that age (OR = 1.063, 95% CI = 1.039–1.088, *p* < 0.001) was a significant independent risk factor for 1-year all-cause mortality. This reminds clinicians to pay particular attention to older patients who have poor prognoses due to increasing years.

To our knowledge, this is the first study to use the MIMIC-III database to construct a prediction model for risk factors affecting 1-year all-cause mortality in elderly patients with cardiac surgery. We used LASSO regression and multivariate regression analyses to screen variables. Subsequently, seven independent risk factors based on the number of cases in the training set were identified, followed by age, sex, SOFA, RR, creatinine, glucose, and RBC transfusion. Finally, all seven predictors were used to develop a proper nomogram prediction model. The model's discrimination and clinical utility were proven using the validation set, and the results indicated that our nomogram model has good clinical application value.

Patients who undergo cardiac surgery have an increased risk of organ malfunction, which indicates a worse prognosis. SOFA score is an objective evaluation system for the severity of organ dysfunction or failure in patients with sepsis, which can scientifically evaluate the condition and prognosis of critically ill patients (17, 18). Cardiac surgery based on cardiopulmonary bypass is frequently time-consuming, severely traumatic, and many patients suffer from organ dysfunction after surgery. Schoe et al. (19) previously showed that the SOFA has good discriminative power for ICU mortality in a cohort of 36,632 patients, with an AUC of 0.865 (0.864–0.866). In addition, Doerr et al. (20) indicated that SOFA is a reliable risk stratification model for ICU death in cardiac surgery patients. In the present study, we found that SOFA is a significant risk factor affecting the prognosis of elderly patients with cardiac surgery (OR = 1.067, 95% CI = 1.008–1.130, *p* = 0.026). For elderly patients who underwent heart surgery, the SOFA score system can be utilized as an early warning index for 1-year all-cause death. Moreover, we found that RR is a crucial prognostic predictor for 1-year all-cause mortality. Patients who died had a significantly higher respiratory rate than those who survived. Overwhelming evidence substantiates that RR is one of the most important physiological indicators of severely ill patients or clinical symptoms worsening (21–23), suggesting that clinicians should identify causes of accelerated respiratory rate as soon as possible and provide individualized treatment.

Interestingly, dysregulated blood glucose metabolism has been linked to adverse clinical outcomes after cardiac surgery (24). In this respect, fluctuating blood sugar levels have been linked to an elevated risk of postoperative complications and lower survival rates after CABG (25). Indeed, rapid blood glucose elevations are common in severely ill patients, not just diabetics. Oxidative stress permeates the onset and development of diabetes and its complications. On the one hand, hyperglycemia can promote the production of free radicals through multiple pathways, leading to oxidative stress (26). On the other hand, oxidative stress can activate multiple mechanisms to cause endothelial dysfunction, which plays a key role in the pathophysiological processes of tissue and organ damage (27). Our findings revealed that glucose levels (OR = 1.013, 95% CI = 1.008–1.017, *p* < 0.001) within the first 24 h of ICU admission represent a major risk factor influencing the 1-year all-cause mortality of elderly patients following cardiac surgery. Clinicians should strengthen postoperative glycemic management and provide treatment based on their previous history and clinical symptoms to reduce the inflammatory state caused by high blood glucose.

Creatinine is a typical indicator of kidney injury in the clinic that has been linked to various other diseases (28). Acute kidney injury is one of the most common and serious complications of cardiac surgery, and it can substantially impact the morbidity and mortality of patients (29, 30). Cardiac surgery-associated acute kidney injury has a complicated pathogenesis, mainly involving inflammatory response, ischemia-reperfusion injury, metabolic disorder, and oxidative stress. Following cardiac surgery, both cardiopulmonary bypass and oxidative damage may boost the inflammatory response, which facilitates a considerable infiltration of immune cells into the renal parenchyma, damaging renal function and lowering patient survival (31). Hou et al. (9) raised the potential of serum creatinine as an outcome indicator in patients with cardiac surgery. The current study revealed that creatinine was a statistically significant factor in predicting 1-year all-cause mortality. An increase in creatinine levels has been shown to alter patient outcomes, which reminds clinicians to monitor the changes of renal function indicators dynamically at an early stage and implement interventions when required.

It is worth mentioning that our study indicated that sex (OR = 0.709, 95% CI = 0.516–0.973, *p* = 0.033) is an independent predictive indicator of cardiac surgery in elderly patients. Women have a worse prognosis after cardiac surgery compared to men. According to a meta-analysis, women who have cardiac surgery are more likely than men to experience a postoperative stroke and short-term death (32). Several reasons can explain this. (1) Female patients more likely to develop diffuse coronary artery disease (32); (2) Women with coronary heart disease have narrower blood vessels, making surgery more difficult; (3) Estrogen may play an important role in the composition of atherosclerotic plaques (33); (4) Related research indicates that women require more annular enlargement since their anatomical annuli are smaller and more technically complex than men's (34); (5) male and female patients may have different valve pathologies (degeneration, calcification, rheumatic, ischemic, etc.), which may affect patient outcomes (35). In addition, we also found that RBC transfusion is associated with poorer outcomes in elderly cardiac surgery patients. This may be attributed to the patients' poor preoperative clinical conditions and the elevated transfusion-related adverse effects (12, 36).

## Limitations

Furthermore, there were some limitations to this research. (1) Given that this was a single-center retrospective study based on the MIMIC-III database, selection bias affected the accuracy of our results to a certain extent. (2) Due to the limitations of the database and the absence of some clinical laboratory indicators, the postoperative serological indicators were not fully included in the construction of the model. In addition, this study mainly focuses on the application of serological indicators and lacks patient comorbidities and imaging examination data. (3) Since the data was gathered more than a decade ago, the decision-making for patients with cardiac surgery changed significantly, which was a confounding factor affecting the clinical outcomes. In the concept of perioperative treatment of cardiac surgery, great progress has been made in nutritional therapy, minimally invasive cardiac surgical operation, ventricular assist devices and other new treatment methods after cardiac surgery. (4) We only evaluated all-cause mortality in the first year following cardiac surgery due to data set restrictions. (5) Because of the limitations of the mimic-iii database, some variables (left ventricular ejection fraction, NYHA, body surface area, and so on) that are required to validate the two-evaluation systems STS score and EUROSCORE are missing. (6) The findings of the present study were only verified internally. Indeed, more research and external validation based on large multicenter cohorts are warranted to increase the robustness of our findings.

## Conclusions

In this large retrospective analysis, our study found that age, SOFA, sex, RR, creatinine, glucose, and RBC transfusion were independent predictors for elderly patients with cardiac surgery. Then, we developed a nomogram that can accurately and reliably predict the 1-year all-cause mortality of elderly patients with cardiac surgery, allowing clinicians to provide personalized treatment and effectively identify patients who will benefit most from surgery.

## Data availability statement

Publicly available datasets were analyzed in this study. This data can be found at the datasets presented in this study can be found in online repositories. The names of the repository/repositories and accession numbers can be found at: https://mimic.mit.edu/docs/iii/tables/.

## Author contributions

TX conceived of the study and drafted the manuscript. QX and XZ design of the study and interpretation of the statistic design. YT gathered and processed the data. HR, CL, and JZ conception, supervision, and revised the manuscript. All authors contributed to the article and approved the submitted version.

## Funding

This study was supported by funding from the National Nature Science Foundation of China (Grant No. 82072145) and the Clinical Research Award of the First Affiliated Hospital of Xi'an Jiaotong University (Grant No. XJTU1AF-CRF-2020-003).

## Conflict of interest

The authors declare that the research was conducted in the absence of any commercial or financial relationships that could be construed as a potential conflict of interest.

## Publisher's note

All claims expressed in this article are solely those of the authors and do not necessarily represent those of their affiliated organizations, or those of the publisher, the editors and the reviewers. Any product that may be evaluated in this article, or claim that may be made by its manufacturer, is not guaranteed or endorsed by the publisher.
